# Combining PARP and DNA-PK Inhibitors With Irradiation Inhibits HPV-Negative Head and Neck Cancer Squamous Carcinoma Growth

**DOI:** 10.3389/fgene.2020.01036

**Published:** 2020-09-10

**Authors:** Ling Zeng, Drexell Hunter Boggs, Chuan Xing, Zhuo Zhang, Joshua C. Anderson, Narendra Wajapeyee, Chris Veale, Markus Bredel, Lewis Z. Shi, James A. Bonner, Christopher D. Willey, Eddy S. Yang

**Affiliations:** ^1^Department of Radiation Oncology, University of Alabama at Birmingham School of Medicine, Birmingham, AL, United States; ^2^Department of Biochemistry and Molecular Genetics, University of Alabama at Birmingham School of Medicine, Birmingham, AL, United States; ^3^O’Neal Comprehensive Cancer Center, University of Alabama at Birmingham School of Medicine, Birmingham, AL, United States; ^4^Department of Cell, Developmental, and Integrative Biology, University of Alabama at Birmingham School of Medicine, Birmingham, AL, United States; ^5^Department of Pharmacology and Toxicology, University of Alabama at Birmingham School of Medicine, Birmingham, AL, United States

**Keywords:** DNA repair, DNA damage, PARP inhibitors, DNA-PK inhibitors, non-homologous end-joining, homologous recombination

## Abstract

Novel targeted agents to inhibit DNA repair pathways to sensitize tumors to irradiation (IR) are being investigated as an alternative to chemoradiation for locally advanced human papilloma virus negative (HPV-negative) head and neck squamous cell carcinoma (HNSCC). Two well-characterized targets that, when inhibited, exhibit potent IR sensitization are PARP1 and DNA-PKcs. However, their cooperation in sensitizing HPV-negative HNSCC to IR remains to be explored given that PARP1 and DNA-Pk_*CS*_ bind to unresected stalled DNA replication forks and cooperate to recruit XRCC1 to facilitate double-strand break repair. Here, we show that the combination of the DNA-PK inhibitor NU7441 and the PARP inhibitor olaparib significantly decrease proliferation (61–78%) compared to no reduction with either agent alone (*p* < 0.001) in both SCC1 and SCC6 cell lines. Adding IR to the combination further decreased cell proliferation (91–92%, *p* < 0.001) in SCC1 and SCC6. Similar results were observed using long-term colony formation assays [dose enhancement ratio (DER) 2.3–3.2 at 4Gy, *p* < 0.05]. Reduced cell survival was attributed to increased apoptosis and G2/M cell cycle arrest. Kinomic analysis using tyrosine (PTK) and serine/threonine (STK) arrays reveals that combination treatment results in the most potent inhibition of kinases involved in the CDK and ERK pathways compared to either agent alone. *In vivo*, a significant delay of tumor growth was observed in UM-SCC1 xenografts receiving IR with olaparib and/or NU7441, which was similar to the cisplatin-IR group. Both regimens were less toxic than cisplatin-IR as assessed by loss of mouse body weight. Taken together, these results demonstrate that the combination of NU7441 and olaparib with IR enhances HPV-negative HNSCC inhibition in both cell culture and in mice, suggesting a potential innovative combination for effectively treating patients with HPV-negative HNSCC.

## Introduction

Current organ preservation treatment strategies for patients with head and neck squamous cell carcinoma (HNSCC) involve concurrent chemoradiation, which enhances radiation (IR)-induced DNA damage. Repair of this damage utilizes either single-strand break (SSB) or double-strand break (DSB) repair pathways. We and others have previously shown that inhibition of poly (ADP) ribose polymerase-1 (PARP1), a member of the SSB base excision repair pathway, is a potent sensitizer of tumor cells to IR (Nowsheen et al., 2011b; Swindall et al., 2013) in HNSCC cells.

Similarly, inhibition of deoxyribonucleic acid protein kinase catalytic subunit (DNA-Pk_*CS*_), a key player in the DSB non-homologous end joining (NHEJ) repair also radiosensitizes cells (Azad et al., 2014; Ying et al., 2016; Brown et al., 2017; Lee et al., 2019). NHEJ is involved in ~80% of DSB repairs induced by radiation in cancer cells (Kakarougkas and Jeggo, 2014), and DNA-Pk_*cs*_ inhibitors, such as the oral inhibitor M3814, can potentiate the antitumor activity of IR in HNSCC cell lines *in vivo* (Zenke et al., 2020).

Previous work shows that PARP1 and DNA-Pk_*CS*_ bind unresected stalled DNA replication forks and cooperate to recruit XRCC1 to facilitate DSB repair (Spagnolo et al., 2012; Azad et al., 2014; Ying et al., 2016; Fok et al., 2019). Additionally, combined inhibition of PARP1 and DNA-PK may increase genomic instability due to differing mechanisms by each inhibitor (Fok et al., 2019). Combination of PARP1 and DNA-PK inhibitors has also been shown to decrease cell growth by 20% *in vitro* and 60% *in vivo* in HNSCC cell lines compared to monotherapy of either agent (Fok et al., 2019). Because unrepaired IR-induced DNA damage may also cause replication stress and mitotic catastrophe (Mahaney et al., 2009), we hypothesized that, due to the crosstalk of these pathways, combining DNA-PK and PARP inhibitors could potentiate IR-induced damage leading to enhanced IR sensitivity in HNSCC cells.

To test this hypothesis, we investigated the *in vitro* and *in vivo* effects of the DNA-PK inhibitor NU7441 and the PARP inhibitor olaparib with irradiation in HPV-negative HNSCC cell lines. Indeed, combining NU7441 and olaparib with IR significantly reduced cell survival compared to IR with either agent alone. Cytotoxicity was due to increased apoptosis and G2/M cell cycle arrest. Mechanistically, kinomic analysis revealed that combination treatment resulted in the greatest inhibition of kinases involved in the CDK and ERK pathways compared to either agent alone. A significant tumor growth delay was observed *in vivo* in UM-SCC1 xenografts receiving IR with olaparib and/or NU7441. These results support the further testing of combining DNA-PK and PARP inhibitors with irradiation in patients with HNSCC.

## Materials and Methods

### Cell Lines and Inhibitors

The HPV-negative UM-SCC1 and UM-SCC6 cell lines were obtained courtesy of Dr. Thomas E. Carey (University of Michigan, Ann Arbor, MI). UM-SCC1-luciferase was obtained from Dr. Eben Rosenthal (Stanford University, Stanford, CA, United States). These cell lines have been previously described (Weaver et al., 2015; Zeng et al., 2017). UM-SCC1 and UM-SCC6 cell lines were maintained in DMEM growth medium (Sigma) supplemented with 10% FBS (SAFC Biosciences) and 1% penicillin/streptomycin (Gibco). The DNA-Pk_*CS*_ inhibitor NU7441 (Tocris Cat #3712) was used at 0.5 μM *in vitro* and 2, 4, and 8 mg/kg *in vivo*. The PARP inhibitors olaparib (LC laboratories Cat #763113-22-0) was used at 3 μM *in vitro* and 25 mg/kg *in vivo*. MK4827 (Selleckchem Cat #S2741), another PARP inhibitor, was used at 100 nM *in vitro*. Cisplatin was used at 4 mg/kg *in vivo*.

### Measurement of Cell Proliferation

Cell proliferation assays were performed as described previously (Weaver et al., 2015; Zeng et al., 2017). Briefly, cells were seeded in 24-well plates and harvested at 72 and 96 h after treatment. Cells were washed with PBS, trypsinized, and diluted 1:20 in isotonic saline solution (RICCA Chemical, catalog #7210-5). Diluted cells were counted using a Beckman Z1 Coulter particle counter. Cell counts were represented as cells/mL.

### Colony Formation Assay

Clonogenic survival was assessed by the colony formation assay as described previously (Nowsheen et al., 2011b, 2012; Zeng et al., 2017). Cells were treated accordingly and remained undisturbed for 2 weeks. Media was not replaced throughout the experiment. Cells were fixed and stained in 25% glutaraldehyde/12 mmol/L crystal violet solution, and the numbers of colonies were counted. Survival fraction was calculated as follows: (number of colonies counted in experimental plate/number of cells seeded in experimental plate)/(number of colonies counted in control plate/number of cells seeded in control plate). A dose-enhancement ratio (DER) was also calculated to illustrate the magnitude of radiation sensitization. The DER is defined as the ratio of the radiation dose required to obtain a surviving fraction (SF) of 0.5, without drug pretreatment, to that required to obtain the same SF after drug pretreatment.

### Cell Cycle

Cell-cycle distribution was measured as previously described (Nowsheen et al., 2011b, 2012; Zeng et al., 2017). Cells were seeded in 100 mm^2^ dishes and treated accordingly. Twenty-four and 48 h after treatment, cells were collected, fixed, treated with RNAse (Sigma, catalog #R-4875), stained with propidium iodide (PI), and read on FACS Calibur using Cell Quest. Data were analyzed using ModFit LT (Verity Software Inc.).

### Measurement of Apoptosis

Apoptosis was analyzed using the Annexin V-FITC Apoptosis Detection kit (BioVision Research Products, 3K101-400) according to the manufacturer’s instructions and was previously described (Nowsheen et al., 2011b, 2012; Zeng et al., 2017).

### Western Blot Analysis

Protein was analyzed by SDS-PAGE as previously described (Nowsheen et al., 2011b, 2012; Zeng et al., 2017). The following primary antibodies from Cell Signaling Technology were used at manufacturer-recommended dilutions for immunoblotting: phosphor-(Thr) MAPK/CDK substrate (#2321), phosphor-erk1/2 (#9101), total erk1/2 (#9102). Actin (Santa Cruz Biotechnology, catalog #sc-47778) was included as a loading control. Species-specific horseradish peroxidase–conjugated secondary antibodies (Santa Cruz Biotechnology) were used at 1:20,000 dilution.

### Kinomic Analysis

Lysates from UM-SCC1 treated with 2Gy IR, with and without 3 μM olaparib and/or 0.5 μM NU7441, were collected immediately after treatment and lysed in MPER lysis buffer with Halt’s protease and phosphatase inhibitors as described previously. After BCA-based protein quantification, lysates were then analyzed with 15 μg of protein on the tyrosine (PTK) arrays and 2 ug of protein on the serine/threonine (STK) arrays as previously described using a PamStation12 (PamGene, The Netherlands) (Jarboe et al., 2012; Anderson et al., 2014; Isayeva et al., 2015). Phosphorylation data was collected over multiple computer-controlled pumping cycles and exposure times (10–200 ms) for ∼144–196 substrates per array. Comparative analysis of kinases upstream of altered peptide prediction was performed in BioNavigator v6.3 using PTK and STK UpKin PamApps (v 6.0).

Whole chip comparative analysis identified that combined olaparib and NU7441 altered kinase activity as compared to IR alone (summarized in Supplementary Table S1). Olaparib- and NU7441-altered kinases were uploaded to GeneGo (portal.genego.com, Clarivate Analytics) to identify biological networks, using indicated maximum node size with an AutoExpand model, canonical pathways, reactions, metabolites, and orphan nodes deselected or excluded.

### Animal Studies

All animal procedures were approved and in accordance with the UAB Institutional Animal Care and Use Committee guidelines. Four-week-old, 20 g, female athymic nude mice (Charles River Laboratories) were allowed to acclimatize for 1 week before experiments. For the orthotopic UM-SCC1-luc model, 100,000 cells were injected into the oral tongue, and tumors were imaged biweekly using a luciferase bioluminescence assay starting at day 4 after injection. Mice received intraperitoneal injections of D-luciferin substrate (150 mg/kg) 15 min before imaging, and luminescence was measured in photons per second. A pilot study was performed to assess potential dose-related toxicities of DNA-PK inhibitor NU7441 (2, 4, or 8 mg/kg IP once daily) in combination with PARP inhibitor olaparib (25 mg/kg, oral gavage twice a day) and irradiation (2 Gy, twice weekly). Treatments were given for three cycles over a total of 15 days. Tumor growth was determined via luciferase, and body weight or any other signs of treatment-related toxicities were recorded. The optimal does of DNA-PK inhibitor NU7441 (4 mg/kg) was selected for the combination treatment in the tumor growth delay study. Cisplatin (4 mg/kg) was also used as a comparison control.

### Statistical Analysis

Data were analyzed by analysis of variance (ANOVA) followed by Bonferroni post-test using GraphPad Prism version 4.02 (GraphPad Software, San Diego, CA, United States). Data are presented as average ± SE.

## Results

### Combining DNA-PK and PARP Inhibition With or Without IR Inhibits HNSCC Growth in Cell Culture

**FIGURE 1:**
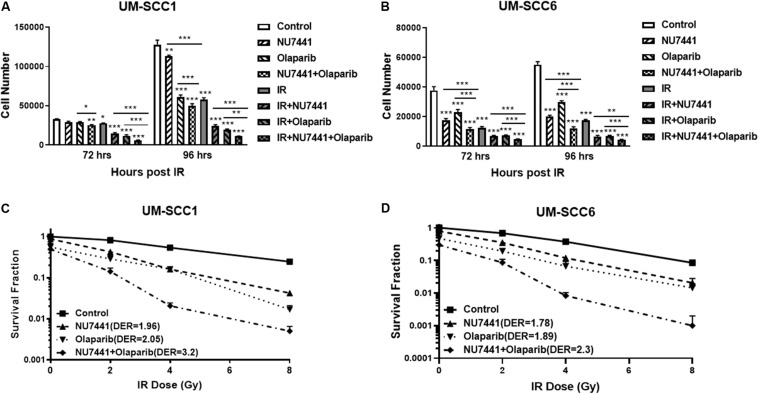
Combination treatment with NU7441 (DNAPKi), Olaparib (PARPi), and IR suppresses **(A,B)** cell proliferation and **(C,D)** clonogenic survival in **(A,C)** UM-SCC1, and **(B,D)** UM-SCC6 head and neck cancer cell lines. For cell proliferation assays, cells were treated with either vehicle or 0.5 μM or 1 μM NU7441 for 16 h, then 3 μM olaparib for 2 h, followed by sham or 2 Gy or 4 Gy IR. Cell numbers were counted at 72 and 96 h after IR using a Beckman Z1 Coulter counter. Shown is the mean ± SEM from at least two independent experiments performed in triplicate; *p < 0.05; **p < 0.01; ***p < 0.001. For clonogenic assays, cells were treated with NU7441 and olaparib accordingly, then followed by 0, 2, 4, and 8 Gy IR. Media was left unchanged for 2 weeks. Cells were fixed, and the number of colonies were counted. Experiments were performed at least in triplicate. Each group showed statistically significant differences (*p* < 0.05).

The potent radiosensitization properties of DNA-PK and PARP inhibitors as well as the interactions of DNA-PK and PARP1 in replication stress repair suggest the potential for increased efficacy by combining these inhibitors with IR. We, therefore, tested the cell proliferation effects of DNA-PK inhibitor, NU7441, and PARP inhibitor olaparib with or without IR in UM-SCC1 or UM-SCC6 head and neck cancer cells. As shown in Figure 1, the combination of NU7441 and olaparib without irradiation significantly decreased proliferation by 60.7% compared to no reduction with either agent alone in UM-SCC1 (Figure 1A) and by 78% in UM-SCC6 (Figure 1B) cells at 96 h. The addition of 4 Gy IR to the combination further reduced cell growth (UM-SCC1: 60.7 vs. 91.3%, *p* < 0.001; UM-SCC6: 78 vs. 92%, *p* < 0.001). To verify the efficacy of this combination, we also performed long-term colony-formation assays. As shown in Figures 1C,D, a 92.2% reduction in clonogenic survival in UM-SCC1 cells was observed (DER = 3.2 at 4 Gy) and 98.8% reduction in UM-SCC6 cells (DER = 2.3 at 4 Gy). Similar inhibition of cell proliferation and inhibition of clonogenic survival was observed with another PARP inhibitor MK4827 (Supplementary Figure S1).

### NU7441 and Olaparib Induce Apoptosis and G2/M Cell Cycle Arrest

**FIGURE 2:**
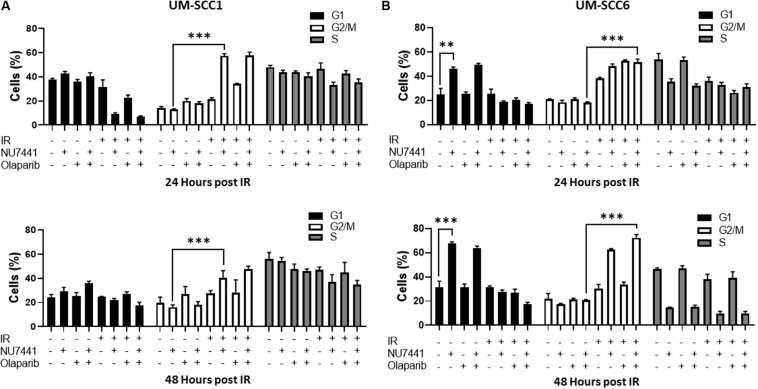
Cell cycle redistribution 24 and 48 h after IR (4 Gy) with NU7441 (DNAPKi) and Olaparib (PARPi) in **(A)** UM-SCC1 and **(B)** UM-SCC6 head and neck cancer cell lines. Cells were treated with either vehicle or 1 μM NU7441 for 16 h, then 3 μM olaparib for 2 h, followed by sham or 4 Gy IR. Cells were stained with propidium iodide at 24 and 48 h after IR and analyzed for cell cycle distribution by flow cytometry. Shown is the mean ± SEM from at least two independent experiments performed in triplicate; **p < 0.01; ***p < 0.001.

One of the major mechanisms of DNA damage-induced cytotoxicity by IR is cell cycle redistribution. Therefore, we next assessed the effects of the various treatments on the cell cycle at 24 and 48 h post IR (4 Gy). At 24 and 48 h post IR, minimal changes in cell cycle distribution were observed with NU7441, olaparib, or IR alone in the UM-SCC1 cells (Figure 2A). Interestingly, combining NU7441 with IR resulted in greater accumulation of SCC1 cells in the G2/M cell cycle compared to NU7441 alone (13.3 vs. 57.4%, *p* < 0.001). However, the addition of olaparib to this combination did not further increase the percentage of cells in G2/M. Similar results were observed with MK4827, which revealed that cells treated with IR accumulate in G2/M, and that is further increased by drug treatment at 12 h post IR. Cells treated with IR alone recover by 24 h post IR although combination groups continue to accumulate in G2/M (Supplementary Figure S2).

In contrast, at 24 h post-IR in the UM-SCC6 cells, NU7441 appeared to cause G1 phase accumulation (25 vs. 46%, *p* = 0.0023, Figure 2B). This effect was further magnified at 48 h post-IR. The addition of IR to NU7441 or olaparib or both NU7441 and olaparib induced G2/M accumulation at 24 h post-IR (18.4 vs. 51.6%, *p* < 0.001) and was further sustained at 48 h post-IR with the triple combination (Figure 2B).

**FIGURE 3:**
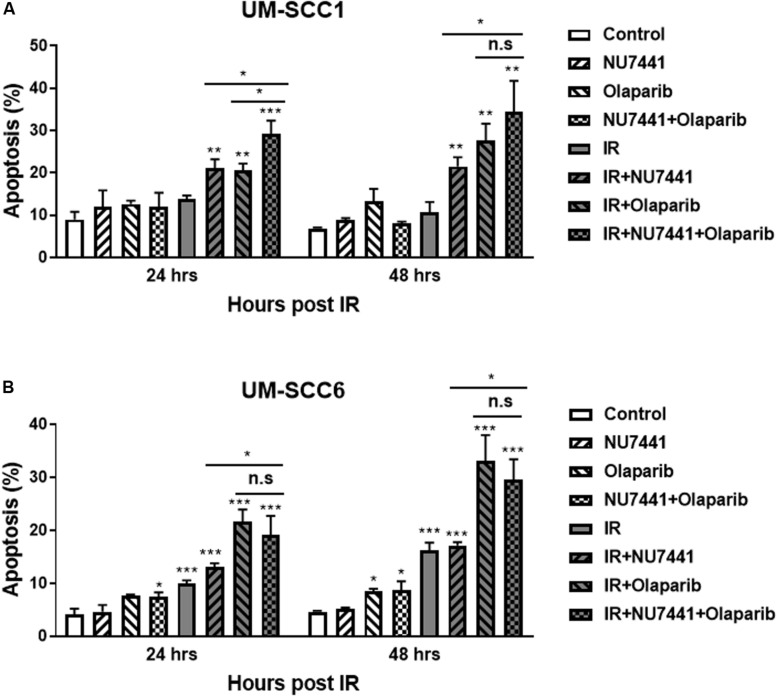
NU7441 and olaparib with IR increases apoptosis in the **(A)** UM-SCC1 and **(B)** UM-SCC6 head and neck cancer cell lines. Cells were treated with vehicle or 1 μM NU7441 for 16 h, then 3 μM olaparib for 2 h, followed by sham or 2 Gy IR. Cells were harvested at 24 and 48 h after IR and evaluated for Annexin V positivity by flow cytometry. Shown is the mean ± SEM from at least two independent experiments performed in triplicate; *p < 0.05; **p < 0.01; ***p < 0.001.

To investigate the effects of NU7441 and/or olaparib with and without IR on apoptosis, we performed annexin V assays. As shown in Figure 3, NU7441 and olaparib alone did not show a substantial increase in apoptosis in the UM-SCC1 (Figure 3A) or UM-SCC6 (Figure 3B) cells. However, in both cell lines, a statistically significant increase in apoptosis was observed at 24 and 48 h with IR in combination with NU7441 or olaparib alone, and that is further increased with the triple combination (UM-SCC1, *p* = 0.003; UM-SCC6, *p* = 0.0001).

### NU7441 and Olaparib Reduce CDK, MAPK, and ERK Signaling

**FIGURE 4:**
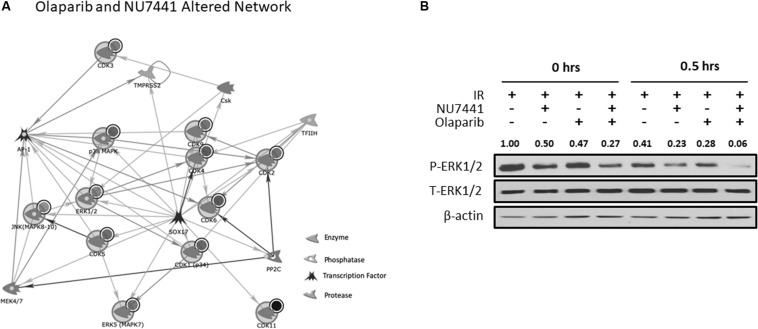
Kinomic analysis following NU7441, olaparib, and IR reveal suppression of CDK and ERK signaling. **(A)** Network of kinases altered by olaparib + NU7441 combination treatment immediately post-IR. Kinases were uploaded by Uniprot ID and overlaid on literature-annotated interactions (portal.genego.com, Clarivate analytics) in a network model displaying centrally effected nodes (AutoExpand < 50 nodes, CDK/ERK centric). Interactions are indicated with lines and arrowheads for directionality. Nodes that are circled indicate input data. **(B)** Phospho- and total ERK status were validated at 0 and 30 min after IR via Western blot. Normalized densitometry values for phosphor-ERK1/2 are also shown.

We and others have previously reported crosstalk between the DNA damage response (DDR) and receptor tyrosine kinase cell signaling pathways (Dittmann et al., 2005, 2008, 2010; Golding et al., 2007, 2009; Nowsheen et al., 2011a, 2012; Jarboe et al., 2012). To perform an unbiased analysis of potential alterations in cell signaling events with our treatments, we performed kinomic analysis using the PamStation12, which allows for real-time detection and kinetic data on kinase/substrate interactions. As shown in Figure 4A, combining NU7441, olaparib, and IR resulted in inhibition of kinases involved in a network centering around CDK and ERK. To validate the kinomic data, we performed western blot analysis in the UM-SCC1 cells treated with various combinations of IR, NU7441, and olaparib. As shown in Figure 4B, the triple combination resulted in the greatest reduction of the levels of phospho-ERK1/2 supporting the kinomic data. The triple combination also suppressed the levels of phospho-MAPK/CDK substrates (Supplementary Figure S3).

### Combination NU7441, Olaparib, and IR Is Well Tolerated and Delays Tumor Growth in HNSCC Xenografts

**FIGURE 5:**
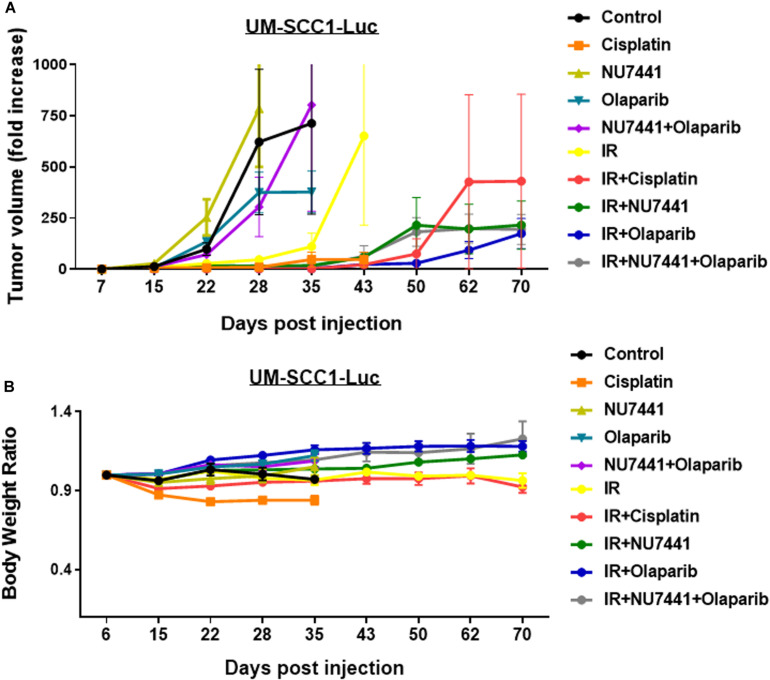
The combination of NU7441 (DNAPKi), olaparib (PARP1), and IR **(A)** reduces *in vivo* tumor growth of UM-SCC1-Luc orthotopic xenografts and **(B)** is well tolerated as measured by body weight. The tongue of athymic nude mice were injected with UM-SCC1 luciferase-expressing cells (UM-SCC1), and tumor volume was measured by bioluminescence imaging twice weekly. Fold changes in each group are shown normalized to luminescence at the start of treatment on day 7. Shown is the mean fold change in tumor volume ± SEM. *N* = 5 mice for all treatment group. **p < 0.01.

To test the *in vivo* effects of NU7441, olaparib, and RT, tumor growth delay was measured using orthotopic tongue HPV-negative UM-SCC1 xenografts. An initial pilot dose-finding study was performed to determine the tolerability and optimal dose of NU7441 to combine with a fixed dose of olaparib (Supplementary Figure S4). As shown in Figure 5A, a significant tumor growth delay was observed in all treatment groups combined with IR (*p* < 0.01). Although not statistically significant, cisplatin-IR trended worse compared to the targeted therapy combinations with IR (*p* = 0.075). Body weight increases were statistically larger with DNAPKi + IR, PARPi + IR, and combination + IR compared to IR alone or IR plus cisplatin, suggesting that combinations of targeted agents with IR is better tolerated compared to cisplatin-IR (Figure 5B).

## Discussion

Since the FDA approval of cetuximab in 2006, no targeted therapeutic combination with IR has been approved for the definitive treatment of HNSCC. Cetuximab, a monoclonal antibody against the epidermal growth factor receptor, is shown to inhibit both NHEJ and HR (Dittmann et al., 2005, 2008, 2010; Nowsheen et al., 2011a, 2012), the 2 major DNA DSB repair pathways. IR-induced DNA damage repair via NHEJ is found to be stimulated by EGFR nuclear translocation and binding to DNA-PK (Dittmann et al., 2005). For HR, EGFR is found to bind BRCA1 (Nowsheen et al., 2011a, 2012). Given the roles of these key DNA repair enzymes in resolution of IR-induced DNA damage, the potent radiosensitizing effects of either the DNA-PK inhibitor or PARP inhibitor in HNSCC is previously reported (Nowsheen et al., 2011b; Forster et al., 2012; Weaver et al., 2015; Kwon et al., 2016; Fok et al., 2019; Lee et al., 2019; Hernandez et al., 2020). DNA-PK inhibition is shown to demonstrate superior radiosensitivity to PARP inhibition in HNSCC cell lines although their combinatorial effect with IR was not tested (Fok et al., 2019; Lee et al., 2019).

PARP inhibition is also shown to inhibit EGFR nuclear translocation following IR, and an induced synthetic lethality is found with combined EGFR and PARP inhibition (Nowsheen et al., 2011a, b, 2012). Recent evidence also reveals a cooperation between DNA-PK and PARP1 at sites of replication fork instability to recruit XRCC1 and coordinate DNA repair at stalled replication forks to effectively protect, repair, and restart stalled replication forks (Spagnolo et al., 2012; Ying et al., 2016). These mechanisms reveal the crosstalk between the EGFR, DNA-PK, and PARP pathways and their putative roles in NHEJ and HR. They also provide the rationale for testing the combination of DNA-PK and PARP inhibition with IR.

Differential effects of DNA-PK and PARP inhibitors on cell cycle distribution are observed between the cell lines. DNA-PK and PARP inhibitors are shown to increase G2/M accumulation (Lee et al., 1997; Carrozza et al., 2009; Jelinic and Levine, 2014; Fok et al., 2019). DNA-PK activity is also essential for resumption of the cell cycle beyond IR-induced G2 checkpoint arrest, and cells exposed to the DNA-PK inhibitor AMA37 demonstrate irreversible G2 accumulation (Sturgeon et al., 2006). We observe a more prominent effect on cell cycle distribution in the UM-SCC6 cells compared to the UM-SCC1 cells, especially a potential senescence-like phenotype in UM-SCC6 cells (Figure 2: increased G1, reduced S at 24 h post IR). Although this is not surprising, due to the heterogeneity of cancer cell lines, the different effects we observe may be due to p53 status. As p53 is an important regulator of the DDR checkpoints (Gadhikar et al., 2013; Dobbelstein and Sorensen, 2015), including the G1/S phase transition, the more pronounced cell cycle redistribution in the UM-SCC6 cells may be due to its wild-type p53 status. Furthermore, it is recently reported that DNA-PK inhibition alone or in combination with PARP inhibition results in accelerated senescence in irradiated cancer cells that is dependent on p53 (Azad et al., 2011, 2014).

Interestingly, kinomic analysis of the combination treatments demonstrates the greatest suppression of CDK and MAPK/ERK pathways. The involvement of these pathways in DNA repair is previously reported (Golding et al., 2007, 2009; Sharma et al., 2007; Khalil et al., 2011; Dean et al., 2012; Zalmas et al., 2013; Wang et al., 2018; Liu et al., 2020). Upon DNA damage, CDK2 activates the DNA damage response, and CDK2 knockout or deficiency increases sensitivity to radiation (Liu et al., 2020). Furthermore, inhibition of CDK4/6 modulates DNA repair (Dean et al., 2012). These actions are likely due to reduced E2F-mediated transcription of DNA repair enzymes (Sharma et al., 2007; Zalmas et al., 2013; Wang et al., 2018). The MAPK/ERK pathways also play key roles in DNA repair (Golding et al., 2007, 2009; Khalil et al., 2011). ERK signaling enhances both NHEJ and HR repair that is dependent on ATM, and blockade of ERK1/2 sensitizes cells to IR. Inhibitors of ERK signaling pathways are shown to block NHEJ-mediated DSB repair as demonstrated through EGFR mutant cell lines by Golding et al.

Our kinomic results also point to potential DNA repair-independent roles of DNA-PK and PARP, as the MAPK/ERK and CDK pathways regulate other cellular processes, including epithelial-mesenchymal transition (EMT). The MAPK/ERK and CDK pathways are implicated in EMT through various mechanisms (Shin et al., 2010) [reviewed in Thiery (2002)]. We have previously reported that, in HNSCC patients, high expression of DNA-PKcs is correlated with recurrence (Weaver et al., 2016). Preclinically, knockdown of DNA-PK in HNSCC cell lines reduces migration and invasion (Weaver et al., 2016). Similarly, DNA-PK is also shown to stimulate tumor cell invasion in head and neck cancer cells with a defective Fanconi Anemia pathway (Romick-Rosendale et al., 2016). A role of DNA-PK and PARP cooperativity in driving ERG-mediated gene transcriptional activation of genes involved in invasion and metastasis is also reported, where the activity of both enzymes is required in these processes (Brenner et al., 2011).

Through its interactions with the cytoskeletal machinery, PARP1 directly regulates cell motility and invasion (Rodriguez et al., 2013; Rom et al., 2016). PARP1 is shown to impact invasion of ovarian cancer cells stimulated by HGF (Wei et al., 2018). In patients with gastric cancer, high PARP1 expression is shown to be associated with increased depth of tumor invasion and lymphatic invasion (Liu et al., 2016). Interestingly, inhibition of PARP reduces motility and invasion of BRAF-mutated melanoma cells (Rodriguez et al., 2013). These results suggest the role of DNA-PK and PARP in EMT, and the connection between DNA-PK, PARP, CDK, and MAPK/ERK pathways may be a mechanism through which the enhanced effects of the triple combination are occurring.

Targeting the DDR has been an attractive strategy in cancer treatment, especially for patients with HR-deficient tumors. In addition to PARP and DNA-PK inhibitors, ATR, CHK1, and WEE1 inhibitors are under development and being tested in current clinical trials alone or in combination with chemotherapy (Brown et al., 2017). Furthermore, combinations of DNA repair inhibitors, such as with PARP and RAD52 combinations, are being developed based on exciting preclinical results (Sullivan-Reed et al., 2018). In this study, we demonstrated that combining DNA-PK inhibition and PARP inhibition with IR in HNSCC results in further reduction in cell proliferation and clonogenic survival. Mechanistically, we show that the triple combination results in the greatest suppression of ERK and CDK signaling that is associated with induced G2/M phase cell cycle accumulation, persistent DNA damage, and increased apoptosis. The results from this study support the testing of this combination with IR in a phase 1b trial as a potential alternative to cisplatin-based chemoradiotherapy to potentially improve the therapeutic index. Combined inhibition of DNA-PK and PARP without radiation is currently being tested in a clinical trial (NCT03907969).

## Data Availability Statement

All datasets generated for this study are included in the article/Supplementary Material.

## Ethics Statement

The animal study was reviewed and approved by the UAB Institutional Animal Care and Use Committee.

## Author Contributions

EY: study conceptualization and supervision. EY, LZ, and DB: study design. LZ, DB, CX, ZZ, and JA: data collection. All authors contributed to data interpretation and manuscript writing.

## Conflict of Interest

EY has served on the advisory board of Astrazeneca, Eli Lilly, Clovis, Strata Oncology, and Bayer and has received honoraria from them. DB received honoraria for speaking engagements and research support for Varian Medical Systems. He also received research support from Novocure. The remaining authors declare that the research was conducted in the absence of any commercial or financial relationships that could be construed as a potential conflict of interest.
