# Supplementary material for: Combining PARP and DNA-PK Inhibitors With Irradiation Inhibits HPV-Negative Head and Neck Cancer Squamous Carcinoma Growth
Source: Front Genet. 2020 Sep 10;11:1036. doi: 10.3389/fgene.2020.01036 (PMC7511754; doi:10.3389/fgene.2020.01036)
Supplement: Supplementary file 1 [file Data_Sheet_1.DOCX]

**Supplemental Figure Legends**

**Supplementary Figure S1**: Combination treatment with NU7441 (DNAPKi), MK4827 (PARPi) and IR suppresses cell proliferation in UM-SCC1 cell line (**A**). Cells were treated with either vehicle or 0.5μM NU7441 for 16 hours, then 0.25μM MK4827 for 2 hours, followed by sham or 2Gy IR. Cell numbers were counted at 96 hours after IR using Beckman Z1 Coulter counter. Shown is the mean ± SEM from one or two independent experiments performed in triplicate; *, *p<0.05*; **, *p<0.01*; ***, p<0.001. NU7441 and Olaparib demonstrate combinatorial dose dependent cytotoxicity in the UM-SCC1 cell line (**B**). Cells were treated with NU7441 and MK4827 accordingly, then followed by 0, 2, 4, and 8Gy IR. Media were remained for two weeks. Cells were fixed and the number of colonies were counted. Experiments were performed at least in triplicate.

**Supplementary Figure S2**: Cell cycle distribution 12, 24 and 48 hours after IR (4Gy) with NU7441 (DNAPKi) and MK4827 (PARPi) in UM-SCC1 cell line. Cells were treated with either vehicle or 1μM NU7441 for 16 hours, then 0.25μM MK4827 for 2 hours, followed by sham or 4Gy IR. Cells were stained with propidium iodide at 12, 24 and 48 hours after IR and analyzed for cell cycle distribution by flow cytometry.

**Supplementary Figure S3**: Phospho-MAPK/CDK substrate levels following various treatment combinations at 0 and 30 min post-IR were assessed via Western blot. Actin was used as a loading control.

**Supplementary Figure S4**: Results from pilot study of treatment toxicity and tumor growth with different concentrations of NU7441 were used in different combinations with olaparib and IR. (**A**) Tumor volume increase with various treatment concentrations tracked for 52 days. (**B**) Body weight with various treatment combinations tracked for 52 days.
