# Supplementary material for: Combining PARP and DNA-PK Inhibitors With Irradiation Inhibits HPV-Negative Head and Neck Cancer Squamous Carcinoma Growth
Source: Front Genet. 2020 Sep 10;11:1036. doi: 10.3389/fgene.2020.01036 (PMC7511754; doi:10.3389/fgene.2020.01036)
Supplement: Supplementary file 2 [file Table_1.DOCX]

**Supplemental Table 1**

Supplementary Table 1 displays Kinases altered by Olaparib+ NU7441 alteration at immediately after treatment (0 min). Kinases are identified by PamGene’s BioNavigator PTK and STK UpKin PamApps (v6.0) and are scored by Mean Final Score (MFS; significance of change) and Mean Kinase Statistic (MKS; amount of change). A negative MKS indicates a decrease in combined Olaparib/DNAPKi treatment reative to control.
