# Supplementary material for: Combining PARP and DNA-PK Inhibitors With Irradiation Inhibits HPV-Negative Head and Neck Cancer Squamous Carcinoma Growth
Source: Front Genet. 2020 Sep 10;11:1036. doi: 10.3389/fgene.2020.01036 (PMC7511754; doi:10.3389/fgene.2020.01036)
Supplement: Supplementary file 3 [file Presentation_1.PPTX]

## Slide 1
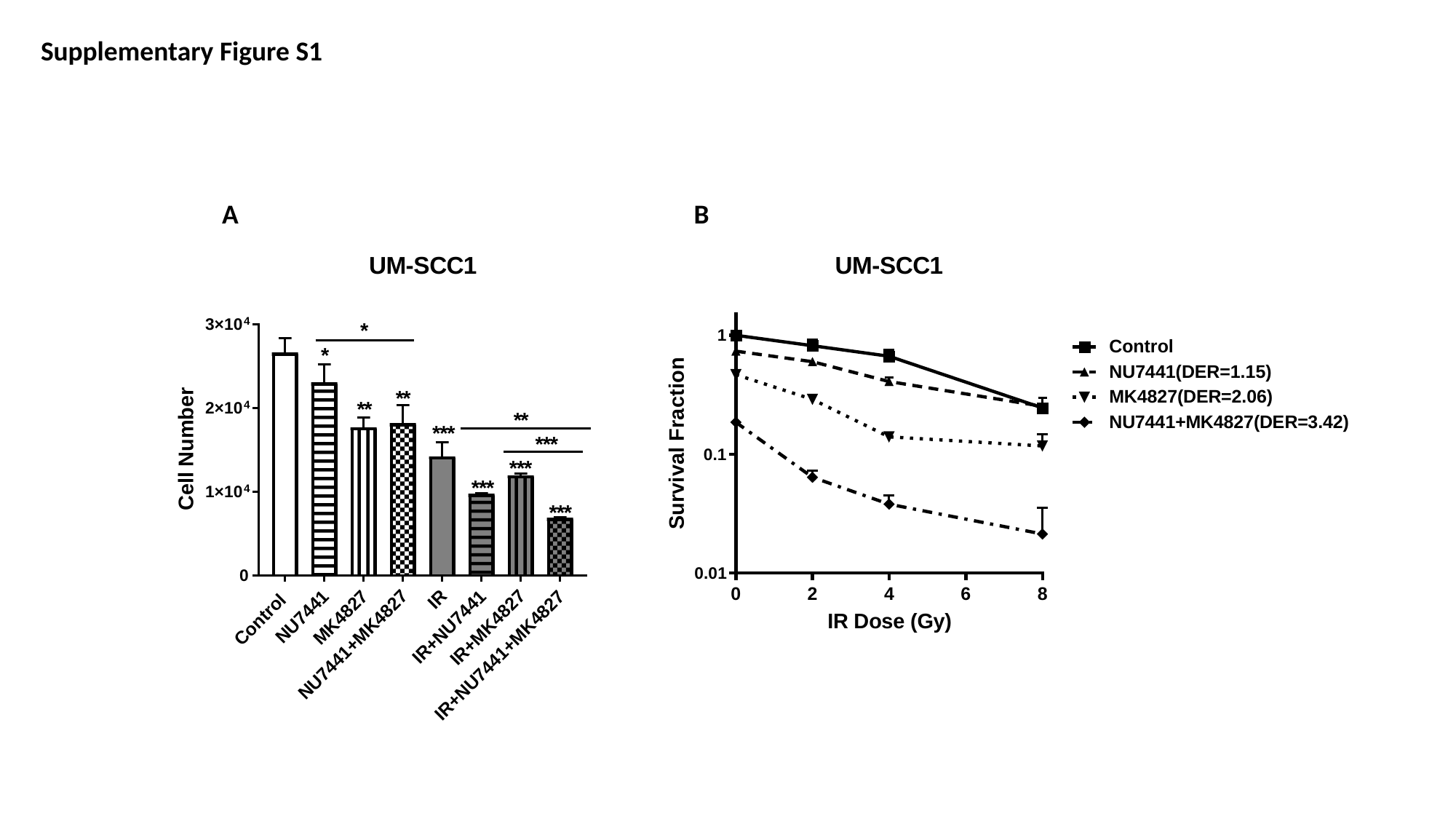

Supplementary Figure S1
A
B

## Slide 2
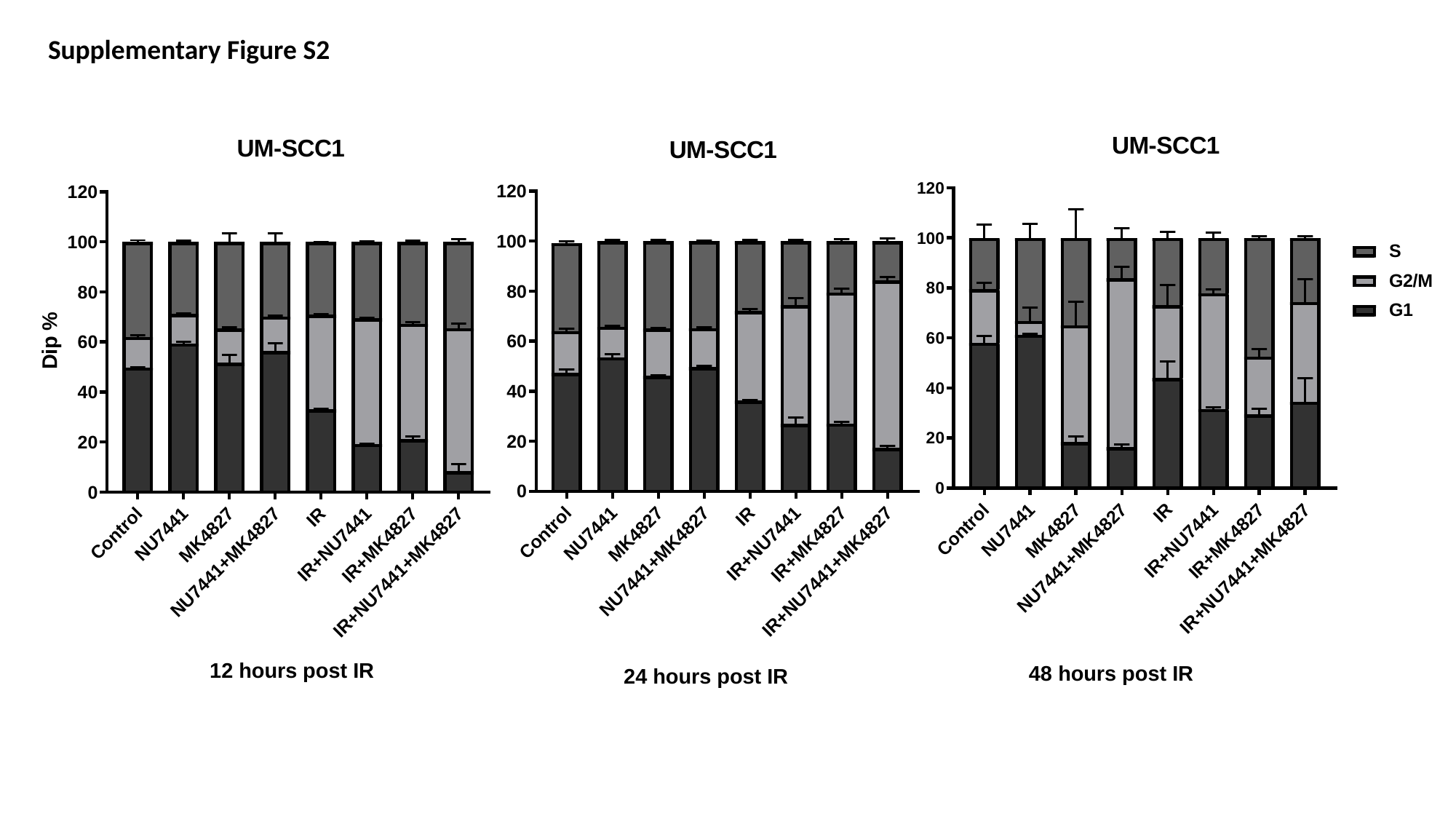

Supplementary Figure S2

## Slide 3
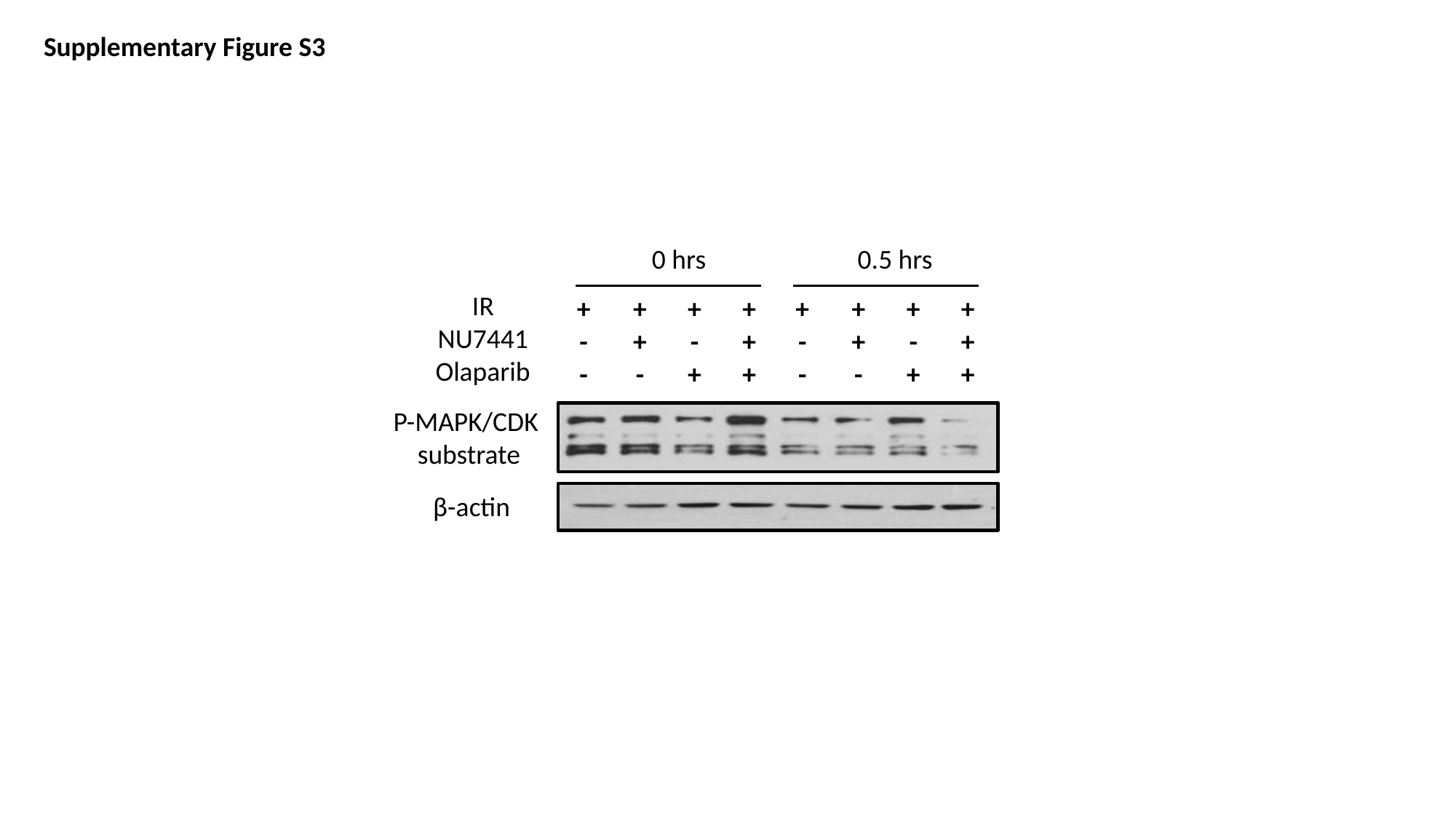

# Supplementary Figure S3
0 hrs
0.5 hrs
IR
NU7441
Olaparib
+
-
-
+
+
-
+
-
+
+
+
+
+
-
-
+
+
-
+
-
+
+
+
+
P-MAPK/CDK
substrate
β-actin

## Slide 4
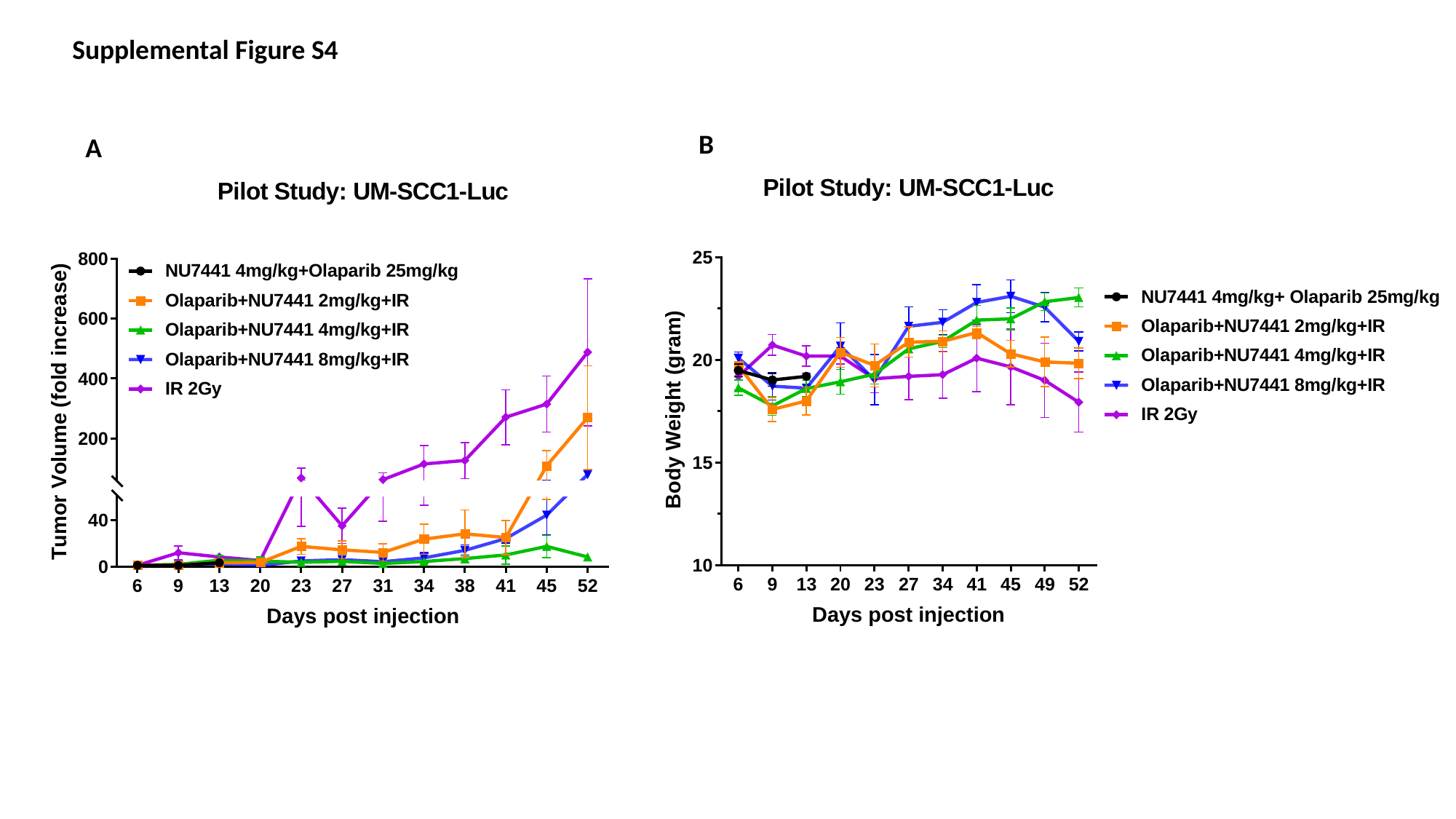

Supplemental Figure S4
B
A
